# Large scale microfluidic CRISPR screening for increased amylase secretion in yeast†

**DOI:** 10.1039/d3lc00111c

**Published:** 2023-07-24

**Authors:** S. Andreas Johansson, Thierry Dulermo, Cosimo Jann, Justin D. Smith, Anna Pryszlak, Georges Pignede, Daniel Schraivogel, Didier Colavizza, Thomas Desfougères, Christophe Rave, Alexander Farwick, Christoph A. Merten, Kevin R. Roy, Wu Wei, Lars M. Steinmetz

**Affiliations:** a European Molecular Biology Laboratory (EMBL), Genome Biology Unit, Heidelberg, Germany; b Lesaffre Institute of Science & Technology, Lesaffre, 59700 Marcq-en-Baroeul, France; c Department of Genetics, Stanford University School of Medicine, Stanford, California, USA; d Stanford Genome Technology Center, Stanford University, Palo Alto, California, USA

## Abstract

Key to our ability to increase recombinant protein production through secretion is a better understanding of the pathways that interact to translate, process and export mature proteins to the surrounding environment, including the supporting cellular machinery that supplies necessary energy and building blocks. By combining droplet microfluidic screening with large-scale CRISPR libraries that perturb the expression of the majority of coding and non-coding genes in S. *cerevisiae*, we identified 345 genes for which an increase or decrease in gene expression resulted in increased secretion of α-amylase. Our results show that modulating the expression of genes involved in the trafficking of vesicles, endosome to Golgi transport, the phagophore assembly site, the cell cycle and energy supply improve α-amylase secretion. Besides protein-coding genes, we also find multiple long non-coding RNAs enriched in the vicinity of genes associated with endosomal, Golgi and vacuolar processes. We validated our results by overexpressing or deleting selected genes, which resulted in significant improvements in α-amylase secretion. The advantages, in terms of precision and speed, inherent to CRISPR based perturbations, enables iterative testing of new strains for increased protein secretion.

## Introduction

The baker's yeast, *Saccharomyces cerevisiae*, has been extensively used to study the secretory pathway^1^ and autophagy,^2^ with applications in both biotechnology and clinical research. Yeast is used to produce high-value products, including pharmaceuticals, antibiotics, antioxidants, vitamins, and recombinant proteins such as insulins, albumins, and antibodies.^3^ The commercial value of recombinant proteins is predicted to double by 2025,^4^ emphasising the current and long-term potential of yeast and other microorganisms as cell factories. Further improvements in the efficiency and yield of recombinant protein production and secretion, will help to reduce the cost and environmental footprint of this industry. An improved understanding of the processes involved in protein secretion also has implications for the treatment of several human diseases in which misfolding of proteins results in aggregate formation and cellular dysfunction.^5,6^

Proteins destined for secretion in eukaryotic organisms are processed through the ER in which nascent proteins are folded and glycosylated, to the Golgi apparatus for further modifications, and packaged into secretory vesicles for delivery to the plasma membrane. The complexity of these processes involving a large number of components and pathways, coupled with system wide metabolic demands for energy and building blocks, suggest that large-scale screens are most suited for the identification of bottlenecks and targets for improvements. Systematic studies of the effects on protein secretion from gene perturbations are challenging.

The secreted protein of interest must be captured for quantification using a method that is amenable to pooled screening. Several recent studies have used microfluidic platforms to identify oversecretion strains in cell populations perturbed by mutagenesis or RNA interference.^7–9^ However, whole genome sequencing is needed to identify the affected locus or loci in mutagenised cells, while RNAi is limited to repressional interference of gene expression, with implications for the speed and scope of discovery.

Here, we perform a systematic interrogation of the effects of gene activation and repression on α-amylase secretion across more than 7000 genes and ncRNAs in yeast. Using large-scale CRISPRi^10^ (repression) and CRISPRa^11^ (activation) libraries, we systematically probe the effects from perturbations of gene expression on α-amylase secretion. The application of CRISPR/Cas9^12^ in combination with high throughput screening and next-generation sequencing (NGS) allowed us to maintain a near genome-wide scope with single gene precision. We identified an increased expression of genes involved in vesicle trafficking, the late Golgi, including retrograde transport from the endosome, and repression of expression of genes involved in the cell cycle and ribosomal processes as beneficial for increased α-amylase secretion. Our results can be applied for yeast strain engineering, to facilitate protein secretion in biotechnological applications, and to improve our understanding of protein processing and secretion in general.

## Methods

### Selection of guide-RNA and oligo design

A single library of guide RNAs for both CRISPRi and CRISPRa, was designed to target promoter regions of 7032 ORFs, SUTs (stable unannotated transcripts), and CUTs (cryptic unstable transcripts), for simplicity, referred to as genes from here on, based on previously published transcription boundaries in *S. cerevisiae*.^13^ For each gene, we identified all potential guide RNAs (gRNA) with minimal predicted off-target effects from 400-bp upstream to 100-bp downstream of the transcription start site (TSS). If there were less than 6 gRNAs in one gene promoter region, we selected all available. Otherwise, we divided the promoter regions into six 50-bp bins, from 300-bp upstream to the TSS. From each bin, the SpCas9 guide with the highest scoring was selected according to the Azimuth prediction software.^14^ This resulted in a library of 40 890 guides (Table S1†) with an average of approximately six guides per feature. The potential for off-target effects was minimised by blasting the individual guide RNAs against each other, as well as all potential gRNA binding sites (4.7 M in total) throughout the genome and removing any guide with less than three mismatches. Oligos were ordered from Agilent using a design that optimises the number of guides per oligo: each 190 bp oligo contains four individual 20 bp guide-RNA sequences interspersed with spacer sequences containing double type II-S recognition sites, enabling restriction digest and release using BspQI with subsequent removal of the recognition site. Example: TCAGTCGATCGgctcttcaaggAAGATA TACGTTATTGATATgttagaagagcgctcttctaggGGAAGGAATATTGA GCAACAgttagaagagcgctcttctaggGCGGGTAACGACAACGAAG TgttagaagagcgctcttctaggTCTCGATTCACCAAACCCT TgttcgaagagcGCTAGCTCCAT.

### Plasmid vectors and background strain

Two plasmids with backbones previously derived^15^ with nuclease-null dCas9 fused to the tripartite VP64-p65-Rta (VPR) domain^11^ for gene activation or the Mxi1 domain^16^ for gene repression were developed for this project. The active domains together with the dCas9 are expressed using a TEF1 constitutive promoter for continuous production of the resulting fusion protein, while the expression of the gRNA is inducible using a TET-On system upon activation by addition of anhydrotetracycline. Selection for the plasmid was maintained using a *kanMX* cassette. Codon optimised α-amylase (AnAmy6) was successfully integrated into the genome of the *S. cerevisiae*, commercially available Ethanol Red background strain at the HO locus with or without Cas9 assisted cutting of the diploid wild-type allele and made homozygous by subsequent loss of heterozygosity. The cassette was integrated with different promoters using a hygromycin selectable marker in two or more copies as estimated using qPCR.

### Plasmid assembly and cloning

Oligos were PCR-amplified using KAPA HiFi polymerase for 20 cycles and the guides released using BspQI restriction digest overnight in CutSmart buffer at 50 °C, followed by Antarctic phosphatase treatment at 37 °C for 30 min and heat inactivation at 80 °C for 2 min. Subsequent reannealing was performed at room temperature. Plasmid vectors were similarly digested overnight at 50 °C using BspQI, followed by a second digest of the released spacer using AscI for the activation plasmid and NotI-HF for the repression plasmid, and clean-up using a Monarch PCR & DNA cleanup kit (New England Biolabs, MA, USA). DNA inserts encoding guide RNAs (10 ng) were mixed with plasmid vectors (0.5 to 1 μg) and concentrated T4 DNA ligase (2 million units per μl) in T4 DNA ligase buffer and incubated at 25 °C for 1 hour before heat inactivation at 65 °C for 10 min. The ligation product was dialyzed for 25 min on Type-VS Millipore membrane against distilled water. Plasmids were transformed using electroporation at 1.8 or 1.9 kV, 200 Ohm, 25 uF into NEB 10-beta electrocompetent *E. coli* cells following the manufacturer's protocol using 2 μl of ligation product per 25 μl of cells. The cells were spread on LB plates with kanamycin added at 30 μg ml^-1^ and incubated overnight after 1 hour of outgrowth at 37 °C. Following outgrowth, the cells were scraped off the plates into LB on ice and centrifuged to collect the cell pellets. The cloned plasmid libraries were extracted using the QIAGEN Plasmid Maxi kit and stored at -80 °C.

### Yeast transformation and harvesting

**Fig. 1:**
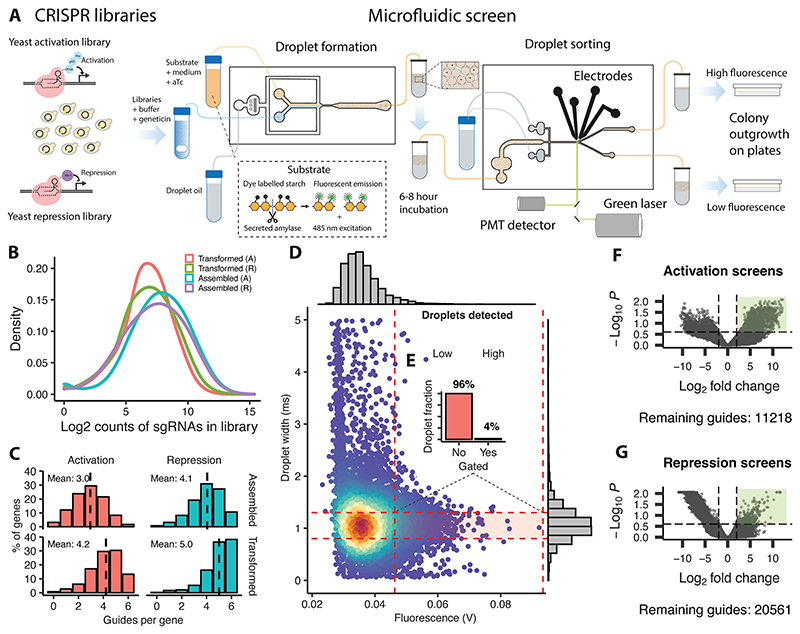
Schematic illustration of the CRISPR based perturbation and microfluidic screening of yeast cells for increased α-amylase secretion, and overview of the assembled libraries, screening, and sequencing of sorted cells. (A) Plasmid-based gene activation and repression libraries were transformed into α-amylase secreting yeast. These libraries were mixed with a fluorescent substrate, growth medium and anhydrotetracycline to induce gene activation or repression and loaded on a droplet forming chip to generate droplets. The droplets were incubated for 6–8 hours at ambient temperature to allow for protein secretion and then transferred to a droplet sorting chip. The droplets were sorted into a high and low fluorescence fraction using electrodes based on the droplet width and the amount of emission from the fluorescent substrate from a single droplet. Sorted cells were released from the droplets and plated for outgrowth before processing for sequencing. (B) Density distribution of guide counts per library during assembly and transformation for activation (A) and repression (R) libraries, (C) histogram showing the average number of surviving guides per gene and the percentage fraction of the total guide pool during assembly and transformation for activation and repression libraries, (D) microfluidic sorting example showing the ratios (E) for the gated fraction (high fluorescence) versus the non-gated population (low fluorescence) fraction based on the droplet width and fluorescent emission during a single round of sorting. Volcano plots of enriched guides (green rectangle) as identified by NGS for the activation (F) and repression screens (G) and the number of remaining guides meeting the filtering criteria, from the statistical analysis comparing counts of guides from the low and high fluorescence fractions from replicate rounds of screening.

The two resulting plasmid libraries (activation or repression) were transformed separately into the background strain using a modified protocol based on Benatuil *et al.^17^* with the addition of 100 μl denatured herring sperm (2 mg ml^-1^) to 400 μl of cells in electroporation reaction buffer to further increase the efficiency. Each reaction contains 400 μl of cells with 5 μg of plasmid libraries. Electroporation was performed 5 at 2.5 kV, 400 ohm, and 25 μF, followed by immediate addition of 1 mL of YPAD and outgrowth for 2 hours at 30 °C at 450 RPM on a shaking heater block. After centrifugation for 2 min at 7000 RPM, the cells were resuspended in 1 M sorbitol and plated on YPAD with sorbitol (1 M) and geneticin (400 μg μl^-1^). The yeast colonies containing the final transformed libraries were collected the next day using a glass cell spreader and the addition of 2 ml of YPAD. The suspended cells were transferred to an Erlenmeyer flask on ice, followed by centrifugation in 50 ml Falcon tubes at 4 °C to pellet the cells. Collected cells were resuspended in 500 μl of YPAD and mixed with 30% glycerol before storage at -80°C. Sequencing of the original assembled and transformed libraries identified a gRNA representation of 72 and 86%, respectively, for the activation and the repression libraries following assembly, and 49 and 69% after retransformation into yeast. The two final yeast libraries with activation or repression plasmid libraries had an average coverage of 3.1 and 4.0 guides per gene (Fig. 1C).

### Microfluidics

The design and manufacturing of PDMS chips and the equipment used for the microfluidic screening is described in Chaipan *et al*. (2017).^18^

### Droplet formation

Yeast cultures with plasmid libraries were inoculated using 50 μl of stock culture in 50 ml of YPAD with geneticin for overnight growth. Cells were collected by centrifugation and washed twice in phosphate-buffered saline (PBS), sonicated for four rounds of 10 seconds on ice, and adjusted to 125 million cells in 5 mL PBS with geneticin (400 μg mL^-1^) to achieve a final cell to droplet ratio of 0.4. Substrate for α-amylase detection from the EnzChek Ultra Amylase assay kit (Invitrogen) was prepared according to manufacturer's instructions and adjusted to 1 mL in PBS before the addition of 4 mL of YPAD and 500 ng μl^-1^ anhydrotetracycline (aTc). The cells with PBS and the substrate in YPAD were loaded on separate 5 ml syringes, with a small bar magnet added to the cell syringe and a 20 μm PES filter added to the substrate syringe. The latter was further covered in aluminium foil to protect the light-sensitive components.

Droplets were generated on a flow focusing droplet generator chip, using flow rates of 400-500 μl per hour for the aqueous phase, and 1100 μl per hour for the oil phase (QX200 Droplet Generation Oil for EvaGreen, Bio-Rad). Once a stable flow had been established, droplets were collected into 1 ml syringes which were then placed on ice and protected from light. The syringes were sealed with parafilm and aluminium foil and stored overnight at 7 °C, then incubated for 6–8 hours at room temperature to allow protein secretion and amylase substrate conversion within the droplets.

### Droplet sorting

The droplets with cells were introduced into the sorting chip at a flow rate of 20 μl per hour and spaced using oil infused at 500 μl per hour. The fluorescence emission of single droplets resulting from excitation using a green laser (488 nm excitation) was measured using a photomultiplier tube. The gain of the signal was increased until individual droplets were resolved. Gating was applied to droplets with the highest fluorescent emission, using a sorting window centred on droplets with an average droplet size as shown in Fig. 1E and adjusted to select the top 2–5% of the total population. Single droplets were sorted by application of 1.5 to 1.7 kV across the electrodes using 3–4 ms pulses of square waves at a frequency of 40 kHz to drive the gated population into a sorting channel.

### Harvesting of cells and plasmid extraction

Collected droplets were mixed with 5–10 μl of the droplet destabilising chemical 1*H*,1*H*,2*H*,2*H*-perfluoro-1-octanol (Sigma) after removal of excess oil and vortexed to break the droplets. The released cells were mixed with a small amount of fresh medium without anhydrotetracycline and streaked on YPAD geneticin plates for overnight growth. Cells were collected from plates in 2 ml of YPAD, pelleted by centrifugation, and processed using a FastPrep vortexing machine with added acid-washed glass beads, followed by plasmid extraction using the QIAprep Spin Miniprep kit (Qiagen).

### Guide-RNA amplification and NGS sequencing

The 20-bp variable guide-RNA region on the extracted plasmids was PCR amplified using Q5 high-fidelity DNA polymerase (NEB) and primers specific to the common flanking regions directly outside the guide-RNA insertion site, with added overhanging sequences resulting in the introduction of 4 to 6-bp barcodes followed by Illumina sequencing adapters on the amplified product. The PCR product from multiple reactions per barcode and sample was mixed and cleaned using Ampure XP beads before sequencing on the Illumina NextSeq500 platform using paired-end 75 nt reads.

### Bioinformatics

Reads were demultiplexed using Je,^19^ aligned using BWA^20^ against padded guide sequences, and finally summarised into a count table using an R script. Statistical analysis was carried out using edgeR.^21,22^ Guide counts were normalised using the RLE method,^22^ with counts from each low and high fluorescence fraction from multiple screens (2 in total for the repression libraries and 4 in total for the activation libraries, each with 2 replicates) treated as replicates (**n** = 4–8). Statistical tests were performed using the glmFit and glmLRT functions which fit generalised linear models and conduct likelihood ratio tests. The Benjamini–Hochberg method^23^ was applied to the *p*-values to control the false discovery rate (FDR). Guides for genes meeting a criteria of [log *FC* > 3, FDR < 0.05] were identified as significantly enriched. The fold coverage for screening was calculated by multiplying the number of screened droplets with the loading efficiency *(λ* = 0.4) and dividing the sum with the original library size.

### Enrichment analysis

The guide with the highest fold-change for an individual gene [log *FC >* 3, FDR < 0.3] was selected as representative and tested for enrichment using R packages gprofiler2^24^ or TopGO^25^ *via* ViSEAGO.^26^ Results from gprofiler2 were FDR corrected for multiple testing errors while TopGO results from Fisher's exact test were instead pruned using the elimination algorithm. Results from both tests with a *p*-value below 0.05 were considered significantly enriched. Gene network analysis was performed using ClueGO^27,28^ and including genes with a log *FC* > 8 from the early activation screen [FDR < 0.1] and the main activation screens [FDR < 0.05], and genes from the repression screens [log **FC** > 3, FDR < 0.15]. Evidence codes from all available sources were used for the statistical testing using a right-sided hypergeometric test with FDR correction (**p**-value < 0.05).

### Validation of α-amylase secretion

PCR-amplified native candidate genes were cloned into plasmid p427-TEF between SpeI and SalI and transformed into the background strain for plasmid-based over-expression. Deletion strains were constructed by golden gate assembly of annealed oligos with gRNA sequences targeting the start and end position of the target gene, into sgRNA expression vector pWS082. The assembled plasmid and Cas9 expression vector pWS173 were linearized using EcoRV or BsmBI and co-transformed with annealed repair fragments, consisting of the joined 60 bp flanking regions of each target gene, which upon successful homology-directed repair, resulted in the deletion of the target gene. The insertion or deletion of the target gene was confirmed by Sanger sequencing. The amount of secreted amylase was measured in cultures grown in YPAD medium after 4, 24 and 48 hours using the Ceralpha method using α-amylase assay kits from Megazyme, Ireland. The relative amount of secreted α-amylase per cell was calculated by dividing the measured amount of secreted α-amylase by the optical density (OD600) as measured during the point of sampling. Baseline expression was measured using the transformed empty vector, or the background strain without gene deletion, for overexpression and deletion strains respectively.

### Statistical analysis

Results reported as significant for the *in vitro* validation are based on the results from the analysis of variance (ANOVA) and the pairwise means from Tukey HSD using the AOV and TukeyHSD functions in R, respectively, together with an interaction model for the two experimental conditions strain and time-point. The normality assumption was tested using the Shapiro–Wilks test, while homogeneity of variances was tested using Levene's or Bartlett's test.

## Results

### A pooled CRISPR/Cas9 screening platform for protein secretion in yeast

We utilised CRISPR with nuclease-null dCas9^29^ to perturb a single gene per cell in a pooled format across the genome, coupled with droplet microfluidic sorting (Fig. 1A) using a previously established, fluorescent α-amylase assay^7,8^ and chip designs.^18^ The commercially available strain Ethanol Red, commonly used to produce bioethanol, was used as the background strain.^30^ We engineered this strain to express α-amylase by insertion of an expression cassette containing the codon-optimised α-amylase gene from *Aspergillus niger* into the HO locus and transformed it with plasmid activation or repression libraries (Fig. 1B and C). The microfluidic system was first used to create droplets containing cells (λ = 0.4) from the transformed protein-secreting strain, together with the fluorescent substrate, growth medium, and anhydrotetracycline (which induces the expression of the guide RNA). These droplets were incubated off chip to accumulate secreted protein, and then reintroduced on a second microfluidic chip for sorting. Degradation of the substrate by secreted amylase relieves quenching of a BODIPY dye conjugate. Single droplets were assayed for secreted α-amylase content, by measuring the fluorescent emission resulting from laser excitation of the unquenched fluorescent dye. Droplets of average size with the 2–5% highest fluorescence signal (Fig. 1D and E) were sorted into a high fluorescence fraction by application of an electrical field, with the remaining droplets passing passively into a low fluorescence fraction. The cells were released from the droplets in fresh medium without anhydrotetracycline and plated out for overnight recovery and outgrowth, followed by PCR amplification of the plasmid guide region. Next generation sequencing of the amplicons allowed us to quantify gRNA abundance, and identify enriched genes, by comparing the guide counts between the fractions.

### Large-scale microfluidic screening reveals regulators of α-amylase secretion

We assessed the potential of the platform by screening and sorting approximately 800 000 droplets, at a 8-fold coverage of the original library size (Table S1†), which among a smaller number of hits, identified guides targeting *PDI1*, an essential ER gene for which overexpression has previously been shown to increase protein secretion^31^ and *NMA2*, part of the NAD^+^ salvage pathway that protects from proteotoxicity by clearing misfolded proteins, and also acting as a chaperone.^32,33^ Encouraged by the results we scaled the screen to 2 million droplets to achieve a 20-fold library coverage, and pooled the results from multiple replicate screens during the statistical analysis. The combined results from multiple replicate rounds of screening identified 11 218 guides from the activation screens (Fig. 1F, Table S1†) and 20 561 guides from the repression screens (Fig. 1G, Table S1†) at sufficient coverage to meet the filtering threshold for the statistical analysis; this represents a 27% and 50% coverage of the total guide pool, resulting in a 83% and 97% representation of all genes with 1.6 and 2.9 guides per gene on average for the activation screens or the repression screens, respectively. The analysis identified 311 guides, out of these remaining guides, mapping to 306 individual genes as enriched [log *FC >* 3, FDR < 0.05] for the activation screens, and a smaller set of 34 guides, mapping to 34 genes as enriched [log *FC >* 3, FDR < 0.05] for the repression screens (Table S1†).

### Network analysis identifies an interplay of processes affecting α-amylase secretion

**Fig. 2:**
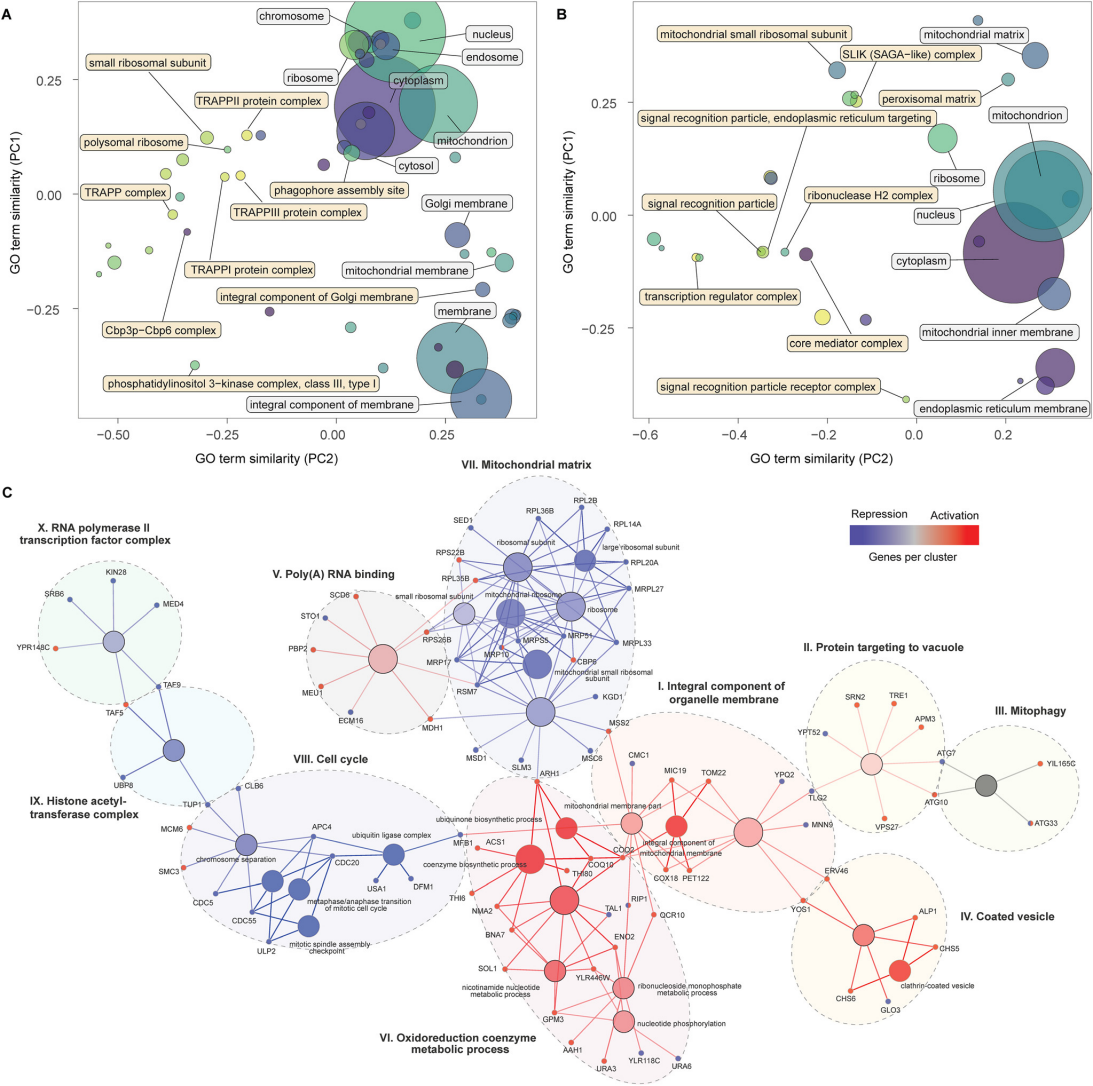
Cellular components, nodes and genes modulating α-amylase secretion. PCA similarity of significantly enriched GO terms for cellular components for the activation (A) and repression screens (B), with terms identified by standard (white) and topology-based (yellow) analysis. The size of the circles represents the number of genes contained in the GO term. Groups of nodes and genes identified as significantly enriched by functional network analysis (C) combining the results from the activation and repression screens. Gene colours, blue (repression) and red (activation), identify the screens for which enrichment was detected, while nodes are coloured in a gradient based on the number of genes from each screen, from red (primarily repression), to blue (primarily activation) and grey (equal). Each cluster is identified by a roman numeral and the assigned name.

To identify cellular processes and pathways that affect α-amylase secretion, we subjected a wider subset [log *FC >* 3, FDR < 0.3] of enriched genes to GO enrichment analysis. Cellular pathways of protein maturation and secretion were enriched (Fig. 2A). These included higher-level terms relating to vesicles, the Golgi apparatus, the periplasmic space, and cell surface for the activation screens. In contrast, for the repression screens (Fig. 2B), the majority of enriched GO terms for cellular components were not directly involved in protein secretion, with the signal recognition particle and the biological process of autophagy (Table S2, Fig. S1†) as exceptions. The screens also identified terms related to the cell cycle, gene regulation and translation, energy supply, biosynthetic processes for sugars, amino acids, and nucleosides as enriched for the activation libraries (Table S2, Fig. S1†). For the repression screens, enriched GO terms included chromosome separation and mitotic division, mitochondrial gene expression and translation (Table S2, Fig. S1†).

Next, we combined a smaller subset of genes (see Methods for details) from the activation and repression screens into a single network, displaying enriched pathways and GO-terms with a FDR < 0.05, in an attempt to provide an integrated map of genes and processes that increase α-amylase secretion. This analysis identified ten clusters (Fig. 2C), six of which were primarily composed of genes from the activation screens (I to VI). Cluster (I) contains genes encoding proteins in the inner membrane of mitochondria, including *COX18* and *PET122*, for which the null mutants are deficient in respiratory growth,^34,35^ and *TOM22*, which is central to protein import into mitochondria during the metabolic switch from fermentative to respiratory growth.^36,37^ Cluster (II) connects *via TLG2*, a gene encoding a t-SNARE protein involved in endosome to Golgi trafficking and the Cvt (cytoplasm-to-vacuole targeting) pathway^38^ together with *YPT52* that enables localization of the CORVET complex to endosomes.^39^ Other genes in this cluster, including *VPS27, TRE1*, and *SRN2*, are involved in the sorting of ubiquitinated proteins for degradation and autophagy (**ATG7**, *ATG10*, including *ATG33* from the mitophagy cluster (III)). The coated vesicle cluster (IV) contains genes linked to COPII transport from the ER to the Golgi, including *YOS1* and *ERV46*, and the two genes in the exomer complex (*CHS5* and *CHS6*) that deliver cargo to the plasma membrane. The poly(A) RNA binding cluster (V) includes *MDH1* part of the malate–aspartate shuttle that exchanges NADH between the cytosol and mitochondria. Overexpression of *MDH1* in *Pichia pastoris* resulted in a 40% increase in recombinant protein production.^40^ This cluster also includes *SCD6* which represses translation in budding yeast,^41^ the ortholog of this gene (tral) in Drosophila is a positive regulator of protein secretion.^42^ The remaining cluster (VI) contains genes associated with ubiquinone biosynthesis *(COQ10, COQ2, ARH1*), as well as *BNA7* and *NMA2* linked to biosynthesis or recovery of NADH and other genes involved in nucleotide-related processes.

The largest cluster of genes from the repression screens encode cytoplasmic and mitochondrial ribosomal subunits (VII). This may be linked mechanistically to another cluster (VIII) containing genes relating to an arrest of the cell cycle. The cell cycle can be indirectly stalled in the G1 phase due to a lack of synthesis of new ribosomes^43^ and directly arrested during multiple mitotic checkpoints *via* cell cycle division (CDC) genes,^44^ several of which are found in this group. Considering that around 30% of the cells energy is allocated towards general protein synthesis during the cell cycle,^45^ a reduction in growth would instead enable shunting of these resources towards synthesis of recombinant proteins. The two remaining clusters (IX–X) included genes that encode components of the histone acetyltransferase complex and transcription factors for RNA polymerase II, highlighting the importance of transcriptional processes. *TUP1* from the first cluster is a general repressor of transcription,^46^ while *TAF5* and *TAF9*, in the interface between the two clusters, are TATA-binding and involved in the initiation of transcription, around 60% of all yeast genes are dependent on *TAF9*, the highest number of dependent genes for all 13 TAF proteins.^47^

### Potential role for non-coding RNAs in modulation of α-amylase secretion

The activation and repression screens identified 71 and 8 SUTs (stable unannotated transcripts) or CUTs (cryptic unstable transcripts) respectively, as enriched. SUTs generate stable transcripts that are thought to interact with other transcripts, while CUTs are unstable and quickly degrade after transcription.^48^ Transcripts of both classes primarily mediate effects on gene expression in *cis*.^49^ An enrichment analysis (see Methods) of genes neighbouring the SUT or CUT (1 kb interval centred on the targeting guide) identified enriched GO terms for the set from the activation screens but not the repression screens. The most overrepresented terms, related to cellular components, contained genes from vacuolar, endosomal and Golgi related processes (Table S2†). Manual inspection of 17 genes found within the top 5 enriched groups of cellular components (Fig. S2†) based on the analysis, out of 102 identified within the range, showed that 15 SUTs or CUTs were situated on the opposite strand, four in a divergent position potentially acting as bidirectional promoters, resulting in gene activation, while eleven were either directly or in a few cases, indirectly through guide interference, overlapping, such that the CRISPRi likely resulted in a simultaneous decrease in the transcription of their antisense gene. Among these, SUT428 overlapping *OPT2* has previously been investigated in detail,^50^ expression of this SUT resulted in a corresponding reduction in transcription of the overlapping gene. The two remaining SUTs or CUTs were found on the same strand, one directly downstream of the identified gene, and the other, CUT727 directly upstream of *BET3*, a core component of the TRAPP complexes. CUTs found upstream, overlapping the 5' transcriptional start site, may act in a regulatory role.^51^ Manual inspection of the small number of enriched SUTs or CUTs from the repression screen (examples in Fig. S3†), showed that CUT361 was situated directly upstream of *ATG19*, an autophagy gene involved in the Cvt pathway. SUT433 and CUT437 were notably situated in tandem, spaced approximately 1 kb apart with SUT433 situated directly upstream of *FLO9*, a flocculation gene, while CUT437 further upstream is in a potential bidirectional promoter position with *GDH3*, involved in glutamate biosynthesis, which has been identified as a target for increased protein secretion both in *S. cerevisiae*^52^ and *P. pastoris.^40,53^ FLO11*, another flocculation gene, which was not identified in this screen, has previously been shown to be regulated by two adjacent ncRNA's.^54^

### Validation of over-secretion genes quantified in the screens

**Fig. 3:**
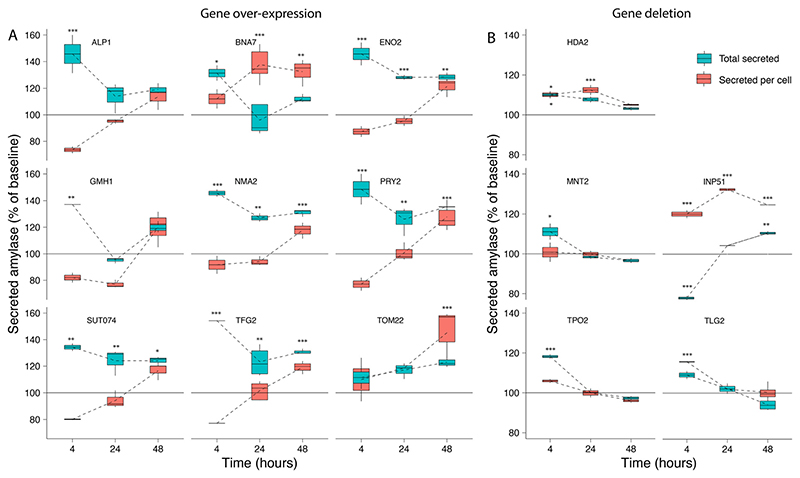
Validation of amylase secretion. For selected genes identified during the activation and repression screens, the corresponding (A) over-expression and (B) deletion strains were generated and assayed for α-amylase secretion over time. The results are shown as total secreted α-amylase (blue) and secreted α-amylase per cell (red) for measurements after 4, 24, and 48 hours, relative to the baseline α-amylase secretion of the transformed empty vector for gene over-expression, or the background strain only for gene deletion. Error bars are one standard deviation from biological replicates *(n* = 2–5), values that are significantly different (ANOVA, *p <* 0.05) from the control (see Methods) within a time point are shown with asterisks corresponding to the *p*-value (***0.001, **0.01 or *0.05) from a Tukey HSD *post hoc* test.

We selected 16 genes identified as significant hits during the screening process for follow-up experimental validation of amylase over-secretion using alternative methods for overexpression or repression. Ten gene hits from the activation screens were validated *via* plasmid-based overexpression of the native gene, while six candidates from the repression screens were evaluated *via* gene deletion in both alleles of the diploid strain. The units of secreted α-amylase and the cell density (OD 600) were measured after 4, 24, and 48 hours of growth (Table S3†) and compared to the baseline expression (see Methods). Overexpression of all genes with the exception of TOM22 resulted in an increase of 35–60% in total α-amylase secretion after 4 hours of growth. This trend continued for *ENO2, NMA2, PRY2*, SUT074 and *TFG2* with 20–40% increases in total α-amylase secretion after 24 and 48 hours (Fig. 3A). Secretion of α-amylase on a per cell basis (see Methods) was significantly increased for *TOM22* in the late stationary phase after 48 hours, and after 24 and 48 hours in BNA7. Gene deletions (Fig. 3B), resulted in increased total α-amylase secretion for *HDA2* (included as a positive control), *MNT2, TPO2* and *INP51* after 4 hours, while for *INP51* a significant decrease was seen after 4 hours, this decrease was not visible after 24 hours and was found to be significantly increased after 48 hours. An increase in secretion of α-amylase on a per cell basis was seen in *INP51* under all time points, in *TLG2* after 4 hours and in *HDA2* after 4 and 24 hours. All over-expressed or deleted genes, with the exception of *TRS20* and *YDR262W* (Fig. S4†), resulted in increased α-amylase secretion under at least one time point.

## Discussion

We applied large-scale CRISPR activation and repression libraries to perturb the expression of the majority of genes and ncRNAs in *S. cerevisiae*, to evaluate the effects on α-amylase secretion. The combination of large-scale CRISPRa/i screening and microfluidics with an alpha-amylase secretion assay adapted to droplet-encapsulated single cells, allowed us to identify 345 genes important for α-amylase secretion. This forms the to date most comprehensive genetic screening effort for α-amylase secretion in budding yeast. The results from activation and repression screens are complementary,^55^ which allowed us to build gene networks and perform enrichment analysis to infer secretion-related cellular processes and molecular machineries from a substantially richer material compared to CRISPRa and CRISPRi alone. We found that increased expression of genes that were previously associated with cellular components linked to the secretory pathway resulted in increased α-amylase secretion, while for gene repression, the majority of enriched components were linked to genes associated with the cell cycle and ribosomal activity.

**Fig. 4:**
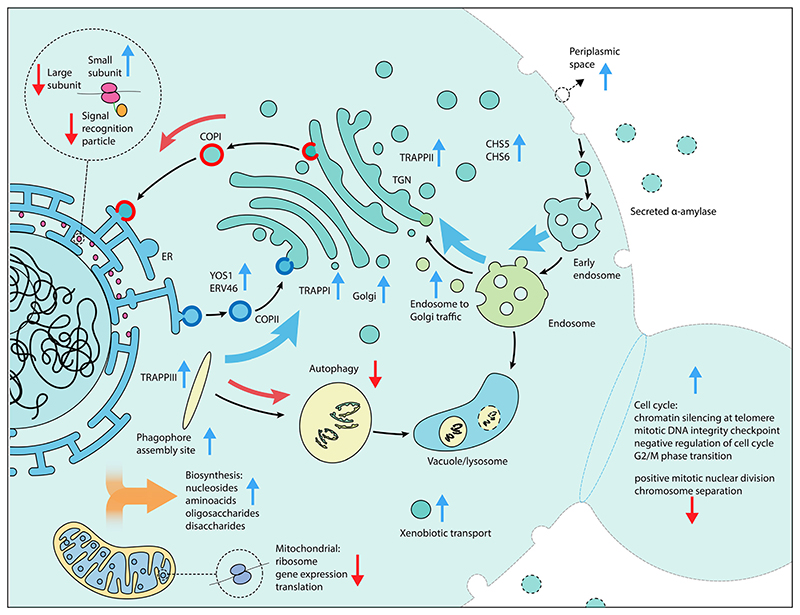
Schematic overview of cellular processes of interest for increased α-amylase secretion. Vertical arrows in blue (gene activation) and red (gene repression) indicate the screen perturbation from which enriched components, functions and genes were detected, while black arrows indicate the direction of traffic between components and curved arrows indicate an increase (blue) or decrease (red) in gene expression as beneficial for increased α-amylase secretion.

Our results emphasise the importance of the cellular complexes and genes that connect the main compartments of the secretory pathway. These include genes involved in retrograde transport from the ER to the Golgi, tethering factors (including TRAPP complexes) that facilitate the capture of the vesicles, and genes that encode proteins that connect the trans Golgi network (TGN), the exomer, and endosome to Golgi transport (summarised in Fig. 4). All three TRAPP protein complexes were enriched in our screen (Fig. 2A). TRAPP I acts in the transport of vesicles from the ER^56^ while TRAPP II acts in intra-Golgi as well as endosome-to-Golgi transport.^57^ Trapp III is similarly involved in autophagy but has also been linked to COPII vesicle formation in mammalian cells.^58^ Secretion and autophagy overlap in a number of processes. COPII vesicles, for which two associated genes (*YOS1* and *ERV46*) were identified as enriched, have recently been shown to be an important source of vesicular membranes for the early enlargement and establishment of the phagophore assembly site (PAS), which initiates the formation of the phagophore.^59^ The term for the PAS was detected as enriched for the activation screens while the term for autophagy was enriched for the repression screen, implying that genes involved in the early formation of the PAS are beneficial for α-amylase secretion but not autophagy in general. Retrograde transport from the endosome to the Golgi was enriched, based on 13 genes, including 5 vacuolar protein sorting genes, in addition to two genes (*CHS5* and *CHS6*) from the exomer complex which acts as a cargo adapter for anterograde transport from the TGN for certain proteins to the plasma membrane.^60^

Among the more unexpected results, we identified components of the signal recognition particle as enriched in the repression screen, suggesting that a reduction in co-translational targeting of nascent protein to the ER is beneficial under some conditions. Balancing the overall metabolic demands of the cell is important. Heterologous expression of recombinant proteins can result in substantial reductions in growth rates due to the metabolic burden.^61^ The results from the activation screens show an enrichment of biosynthetic processes for sugars, including disaccharides and oligosaccharides, amino acids from the aspartate and lysine family, and nucleosides. Biosynthesis of amino acids and translation are the most energetically costly steps that are related to protein secretion.^62^ We further found an enrichment of processes related to NADPH regeneration and shunting which likely help to offset some of the associated energy demand (Table S2†). The activation screen identified negative regulation of the cell cycle *via* DNA-dependent DNA replication and mitotic DNA damage checkpoint and mitotic G2/M transition checkpoint as beneficial, in conjunction with suppression of chromosome separation, localization to the spindle body and positive mitotic nuclear division for the repression screen. Among these genes were *ENO2*, for which over-expression has previously been shown to result in G1 arrest.^63^ Overexpression of this gene as part of our validation, resulted in increased amylase secretion (Fig. 3A). Together, these results suggest that an arrest of the cell cycle is beneficial for increased α-amylase trafficking and secretion. Additionally, the enrichment of genes in the small subunit of the mitochondrial ribosome and mitochondrial gene expression and translation in the repression screen also suggests that a temporary reduction of mitochondrial translation processes is beneficial for increased α-amylase secretion (Fig. 2B).

In our work, we also investigated the influence of long non-coding RNAs on α-amylase secretion. We identified enriched SUTs and CUTs in the vicinity of genes that encode for components in the interface of the late Golgi, endosome and vacuole. Of the manually inspected hits (Fig. S2†), the vast majority were situated on the opposite strand in a position that may result in interference of gene transcription.^64,65^ Notable examples in addition to the previously discussed SUT428 and CUT727 are CUT586 and CUT480. CUT586 overlaps *YPT32* on the opposite strand; *Ypt32* forms a GTPase with *Ypt31*, and regulates the exocytic pathway.^66^ The TRAPPII complex, of which we found several components being enriched in our screen, in turn acts as the guanine exchange factor that activates *Ypt31/ Ypt32.^67^* CUT480 overlaps with *ARF2*, a functionally interchangeable homologue of *ARF1*,^68^ a GTPase with a closely related role to *Ypt31/Ypt32*, which further stimulates *SEC7* during sorting and localization to the late Golgi.^69^ Further among the hits are CUT276, situated opposite of *VPS33*, one of the four core subunits of the HOPS and CORVET tethering complexes. The exact role and mechanisms of action of the identified SUTs and CUTs would need to be experimentally tested. Secondary effects, from guides within distance of the TSS, or overlapping the CDS of other genes, may affect the results.

We successfully used industrial production strains for genetic screens, instead of established laboratory strains. Efficient protein secretion is a valuable trait for biotechnological protein production and the use of a robust industrial strain is beneficial for the interpretability of hits that might be relevant to commercial applications. Performing genetic screens in industrial strains, however, is challenging, since industrial strains are difficult to transform, with reported efficiencies 2 to 3 magnitudes lower compared to established laboratory strains.^70^ This makes the use of these strains for genetic screens challenging since the assembly and transformation of the gRNA libraries has to be carried out at sufficient scale, estimated at around 20 times the library size,^71^ to retain the diversity of the original gRNA pool. Our work confirmed low transformation efficiency. Substantial optimization of strain transformation (see Methods section) allowed us to overcome this issue, which will be useful for future studies within industrially relevant strains. The *in vitro* validation of a subset of enriched genes from the secretion screens supported our findings from the pooled genetic screens (Fig. 3). In summary, over-expression of genes *(ENO2, NMA2, PRY2, SUT074, TFG2)*, or gene deletion (*INP51*) resulted in around 20–30% increases in total α-amylase secretion, sustained over 24 and 48 hours. With higher increases (20–40%) on a per cell basis (*BNA7*, *TOM22*, *INP51)*. The majority of the validated genes have, to our knowledge, not previously been identified as targets for increased α-amylase secretion.

We anticipate that this study can serve as a blueprint for future investigations into the effects of gene expression perturbation as well as gene editing screens on protein secretion. The use of a CRISPR/dCas9 system with guides acting as barcodes, combined with high throughput microfluidics, substantially increases the speed and scale of discovery compared to previous studies.^7–9^ Based on the time requirements of our large-scale screens with tens of thousands of perturbations, which were typically performed over a week, with one day of screening. We believe that smaller screens using targeted libraries, would be able to generate results of similar or higher quality in a matter of days, including transformation, screening and sequencing. This would enable iterative testing and optimization of new strains for improved protein secretion. Future developments could also include the use of CRISPR-based precision genome editing, such as MAGESTIC,^72^ which would allow for probing of natural variants in relevant strains with desirable traits.

## Supplementary Material

ESI 1

ESI 2

ESI 3

ESI 4

ESI 5

ESI 6

ESI 7

ESI 8

## Data Availability

Raw and processed sequencing data are available at Gene Expression Omnibus (GEO) under accession number GSE221655.
