# Supplementary figures and images for: Large scale microfluidic CRISPR screening for increased amylase secretion in yeast
Source: Lab Chip. Author manuscript; Available in PMC 2023 Aug 15. (PMC7614956; doi:10.1039/d3lc00111c)

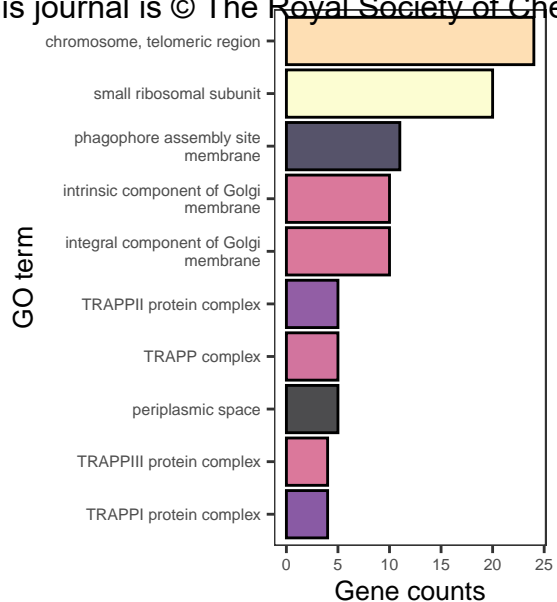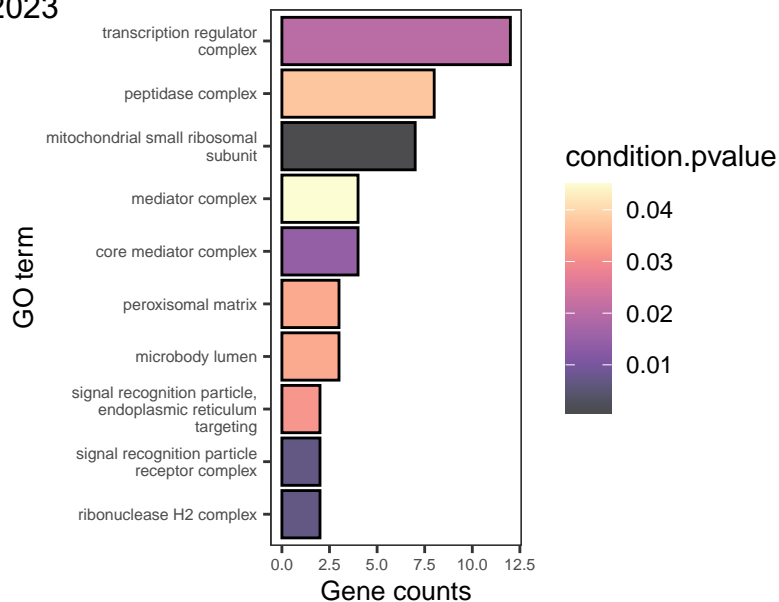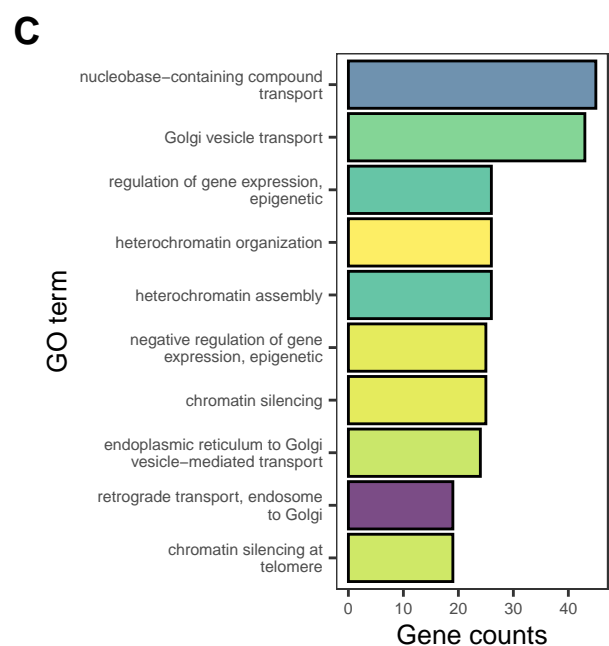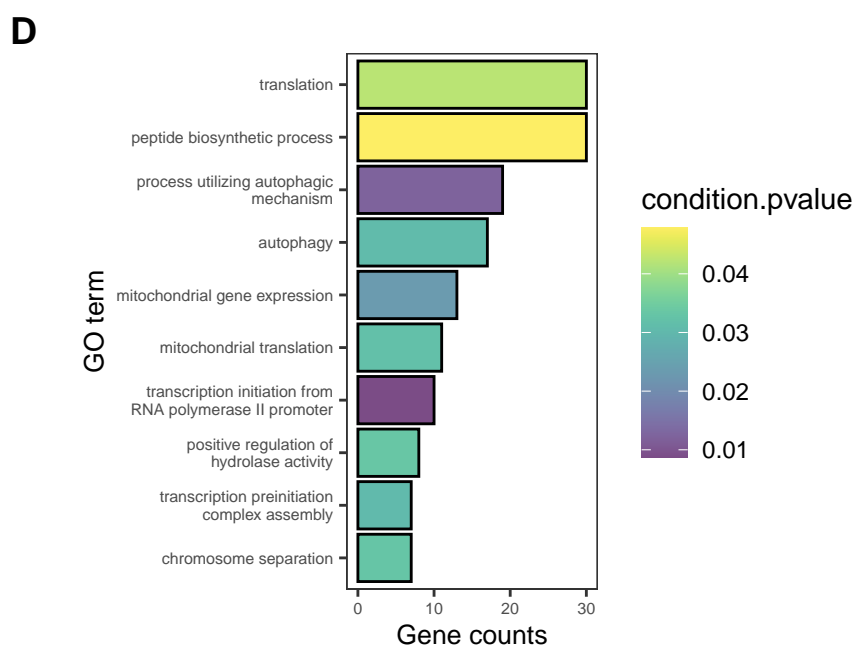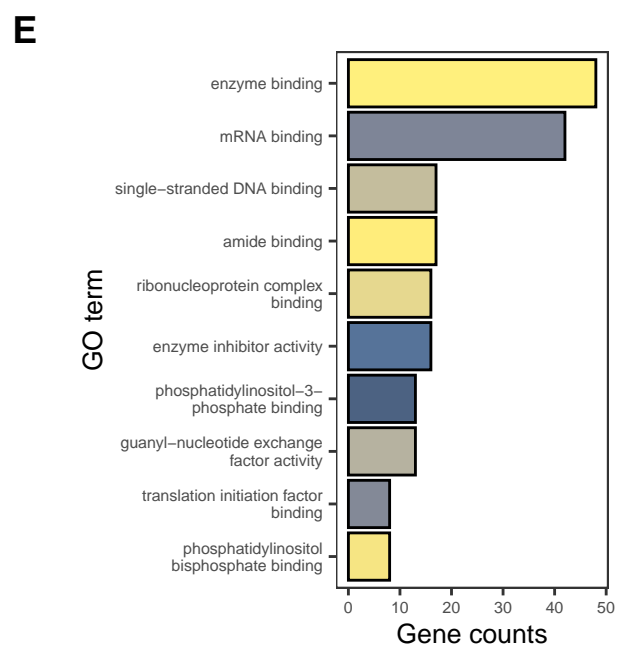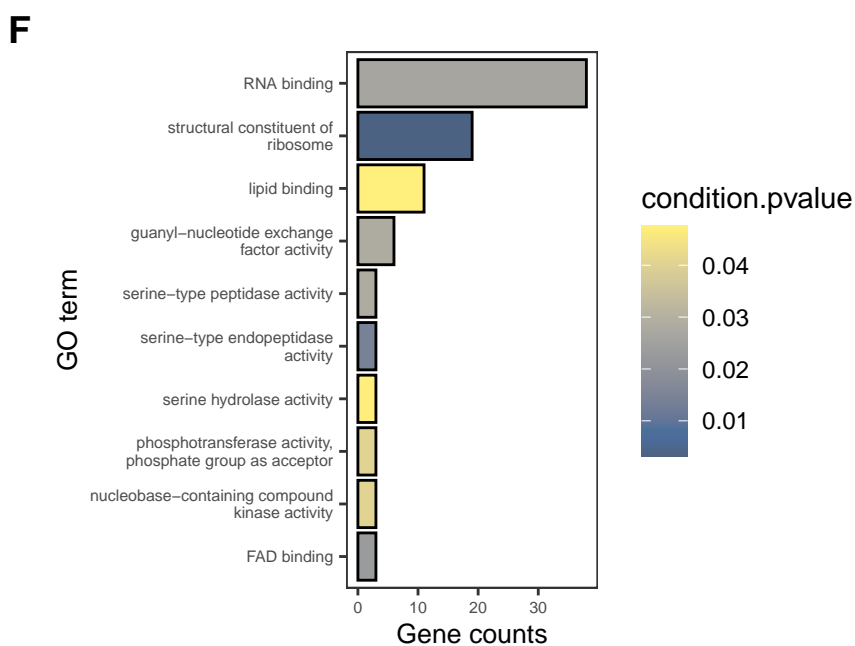

Supplement: ESI 1 [file EMS184631-supplement-ESI_1.pdf]

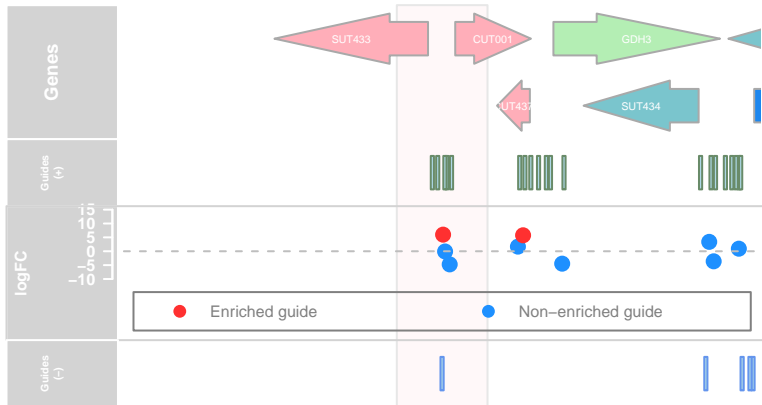

## CUT361 on chr15

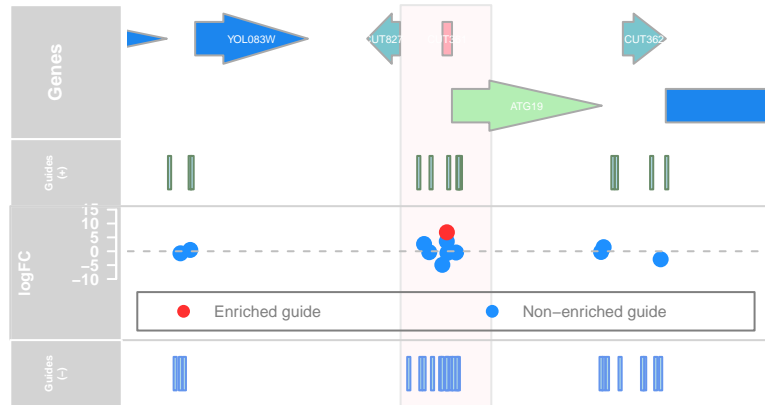

Supplement: ESI 3 [file EMS184631-supplement-ESI_3.pdf]

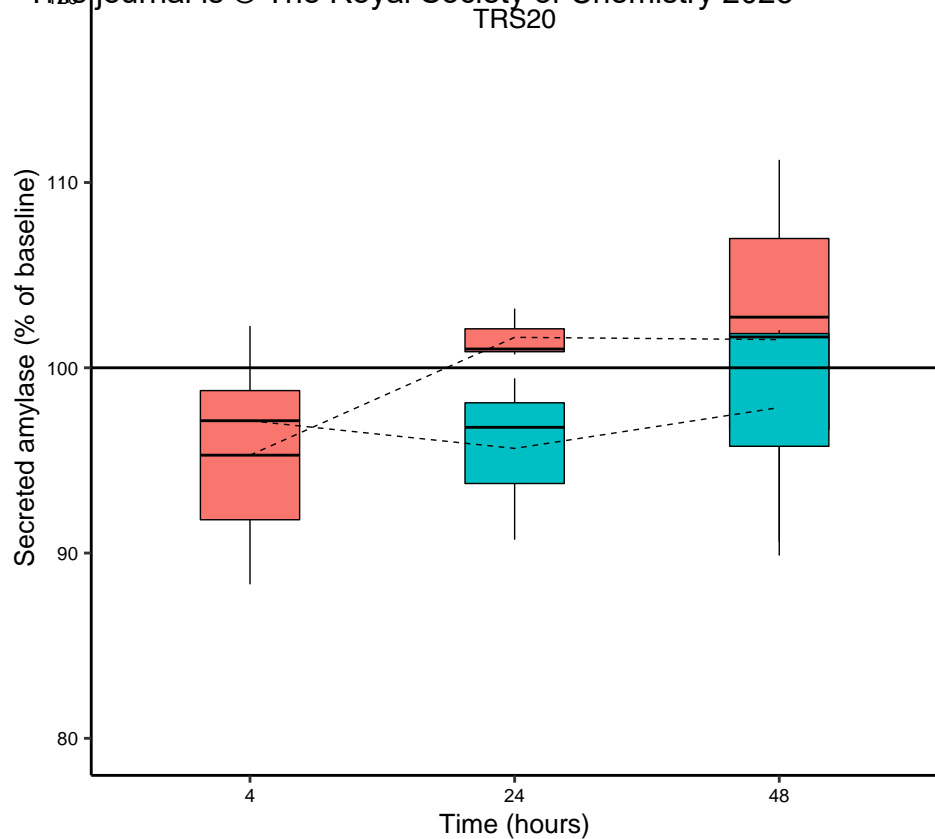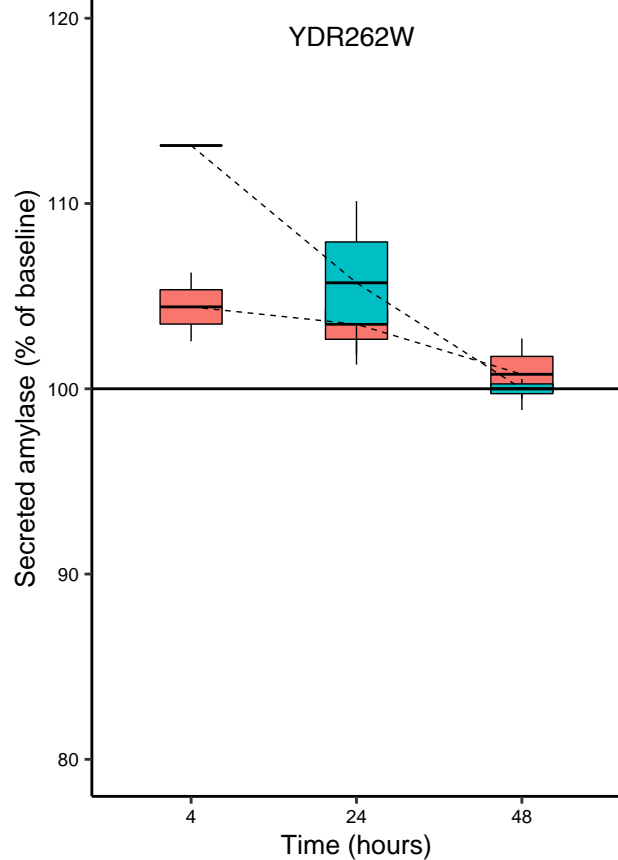

Supplement: ESI 4 [file EMS184631-supplement-ESI_4.pdf]
