# Supplementary material for: Large scale microfluidic CRISPR screening for increased amylase secretion in yeast
Source: Lab Chip. Author manuscript; Available in PMC 2023 Aug 15. (PMC7614956; doi:10.1039/d3lc00111c)

Electronic Supplementary Material (ESI) for Lab on a Chip.
This journal is © The Royal Society of Chemistry 2023

SUT460 on chr4

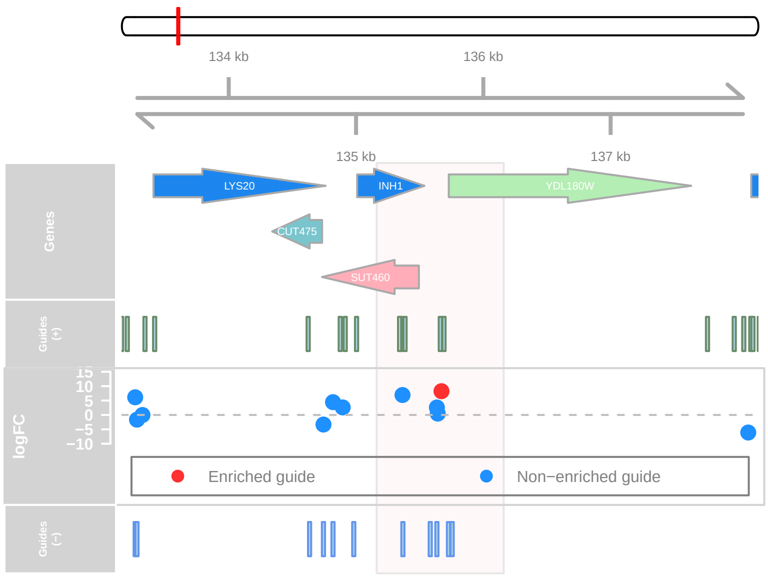

CUT598 on chr7

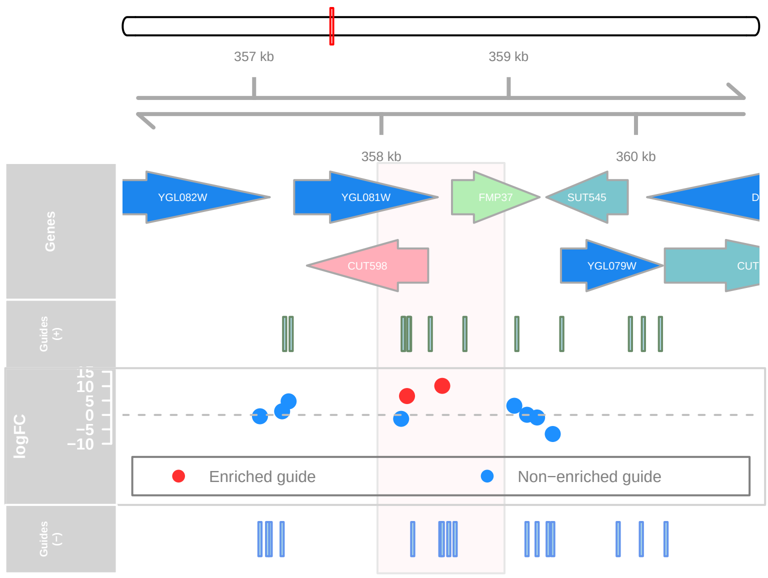

SUT573 on chr8

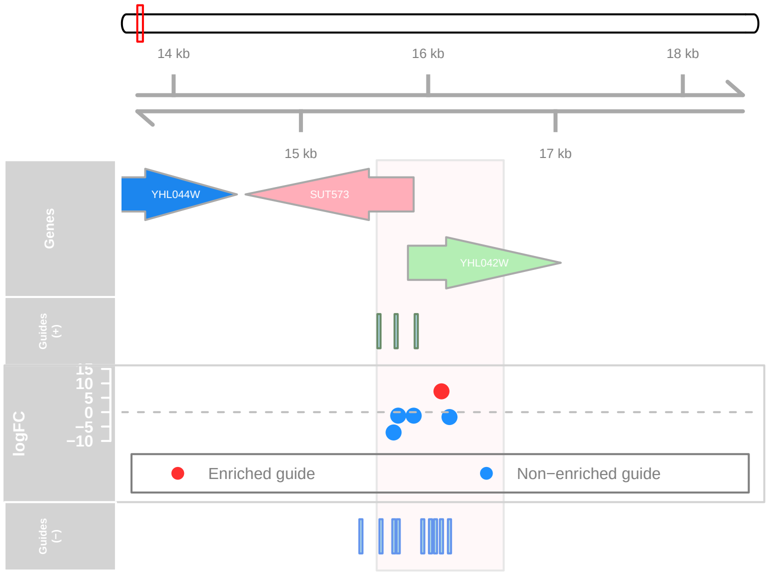

CUT643 on chr8

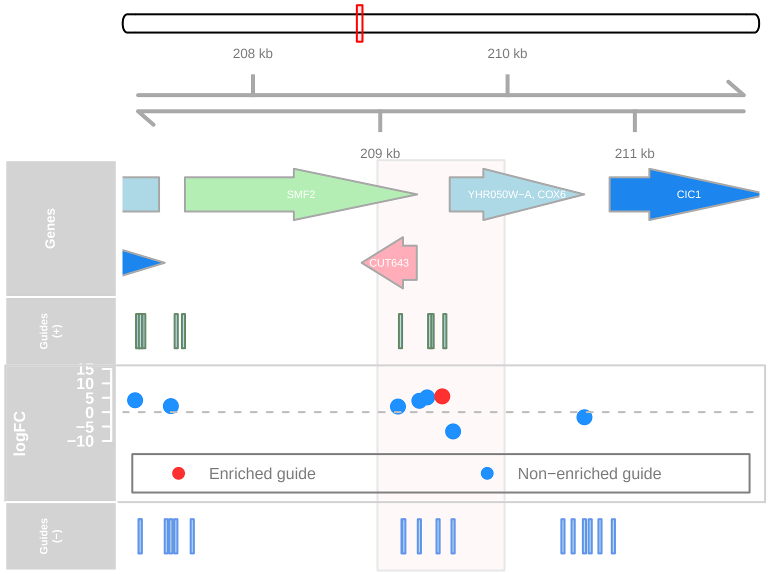

CUT213 on chr10

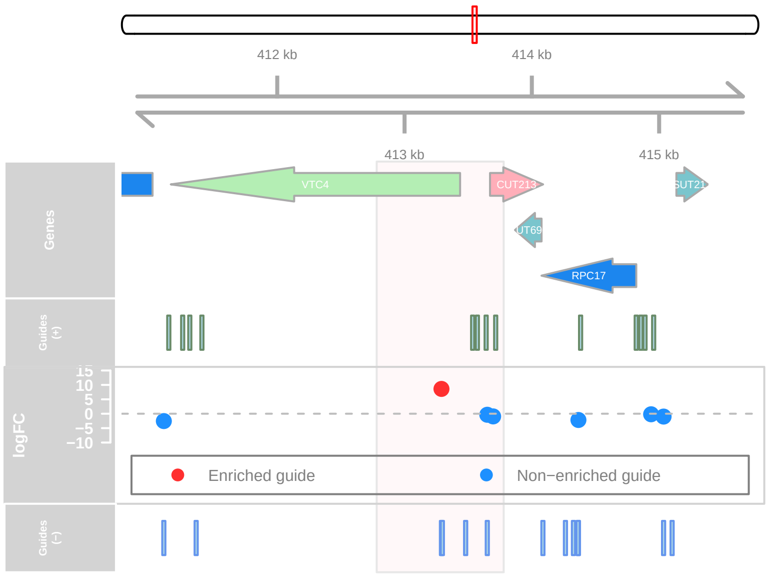

SUT251 on chr12

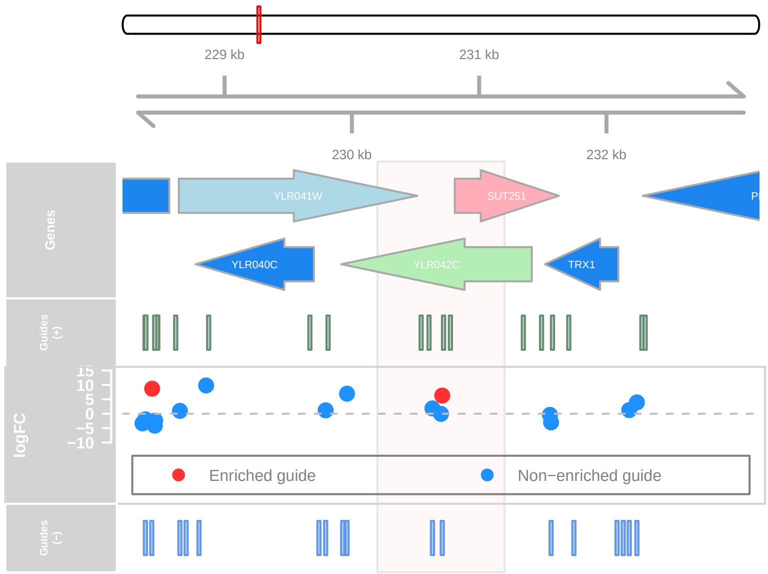

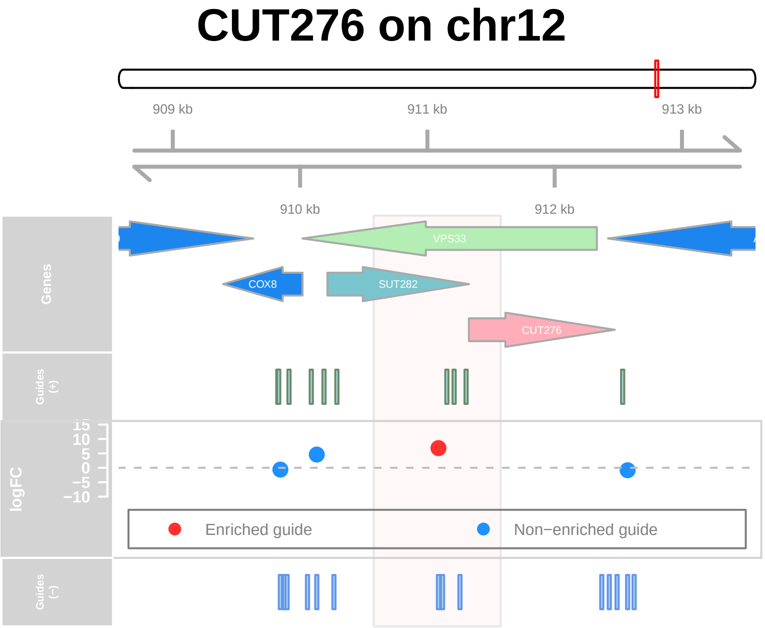
CUT276 on chr12
909 kb
911 kb
913 kb
910 kb
912 kb
Genes
VPS33
COX8
SUT282
CUT276
Guides (+)
logFC
15
10
5
0
−5
−10
Enriched guide
Non−enriched guide
Guides (-)

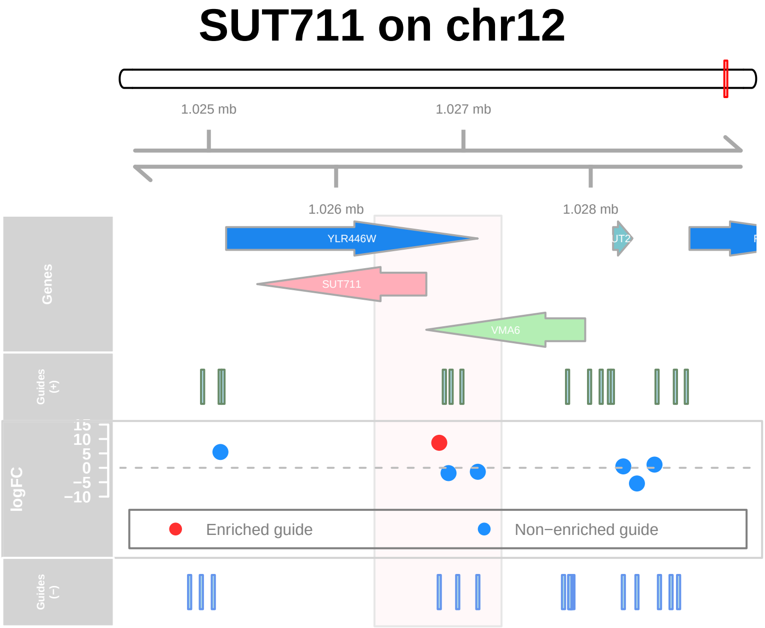
SUT711 on chr12
1.025 mb
1.027 mb
1.026 mb
1.028 mb
Genes
YLR446W
SUT711
VMA6
Guides (+)
logFC
15
10
5
0
−5
−10
Enriched guide
Non−enriched guide
Guides (-)

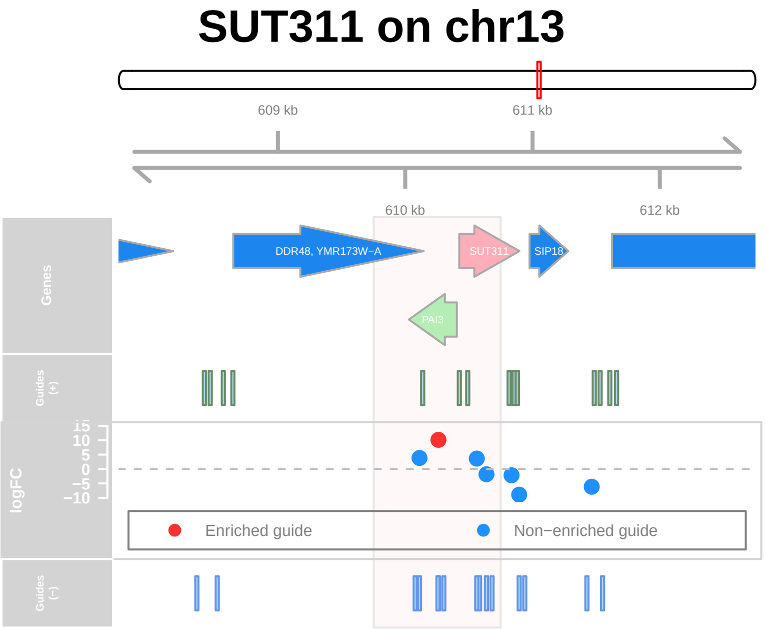
SUT311 on chr13
609 kb
611 kb
610 kb
612 kb
Genes
DDR48, YMR173W−A
SUT311
SIP18
PAI3
Guides (+)
logFC
15
10
5
0
−5
−10
Enriched guide
Non−enriched guide
Guides (-)

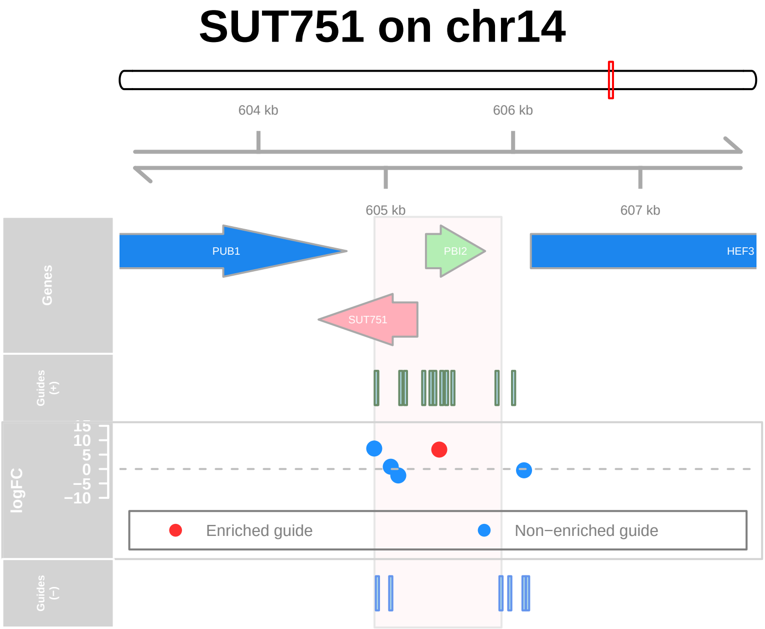
SUT751 on chr14
604 kb
606 kb
605 kb
607 kb
Genes
PUB1
PBI2
HEF3
SUT751
Guides (+)
logFC
15
10
5
0
−5
−10
Enriched guide
Non−enriched guide
Guides (-)

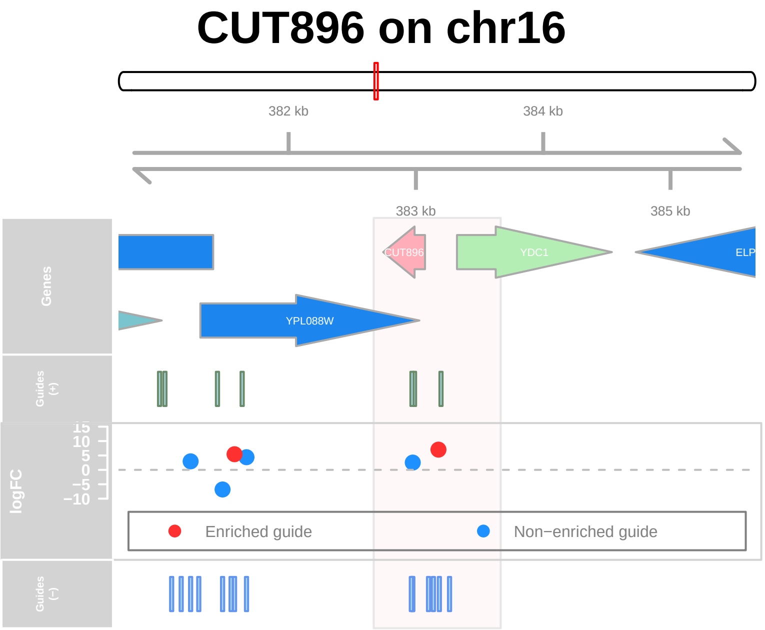
CUT896 on chr16
382 kb
384 kb
383 kb
385 kb
Genes
CUT896
YDC1
YPL088W
Guides (+)
logFC
15
10
5
0
−5
−10
Enriched guide
Non−enriched guide
Guides (-)

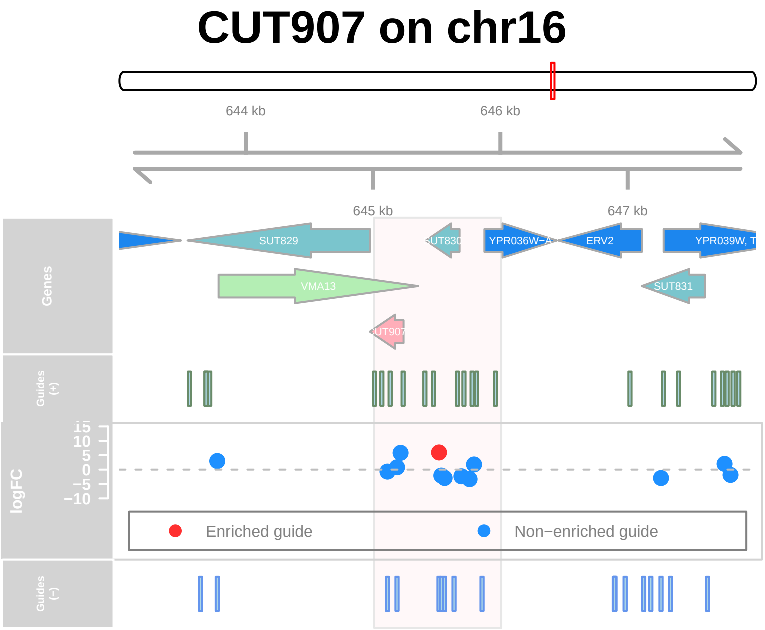
CUT907 on chr16
644 kb
646 kb
645 kb
647 kb
Genes
SUT829
YPR036W−A
ERV2
VMA13
SUT831
Guides (+)
logFC
15
10
5
0
−5
−10
Enriched guide
Non−enriched guide
Guides (-)

SUT428 on chr16

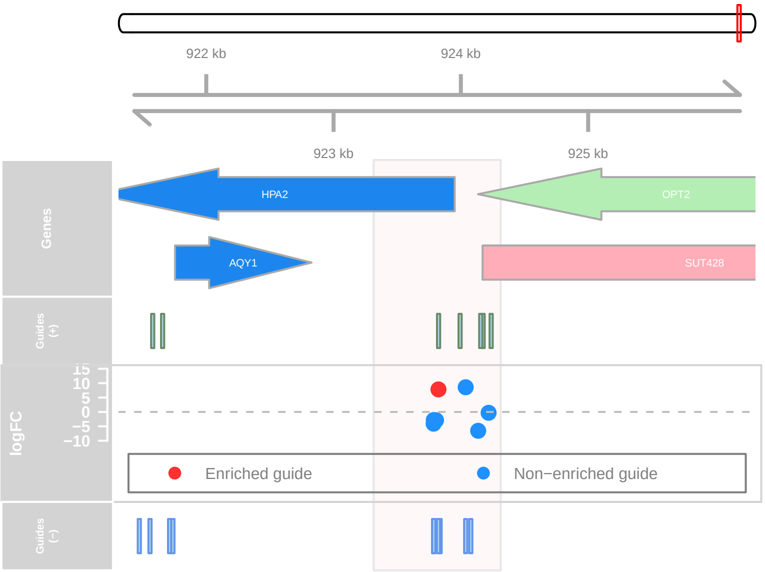

CUT586 on chr7

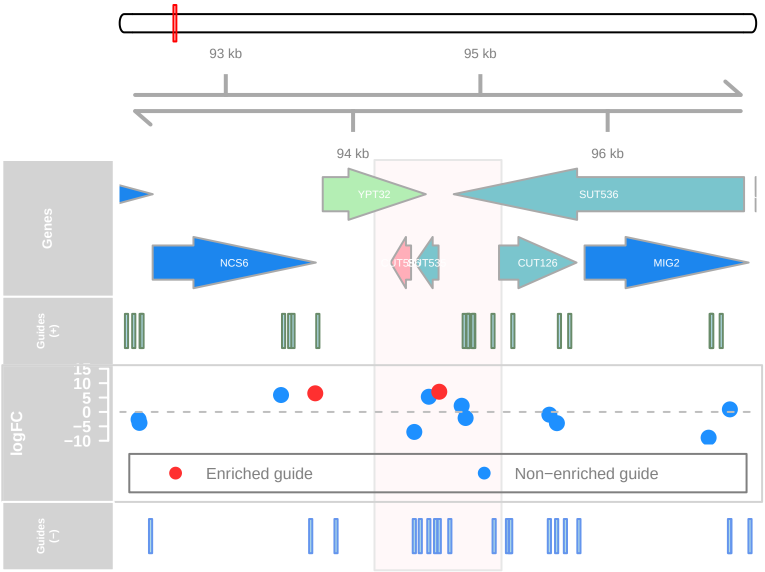

CUT727 on chr11

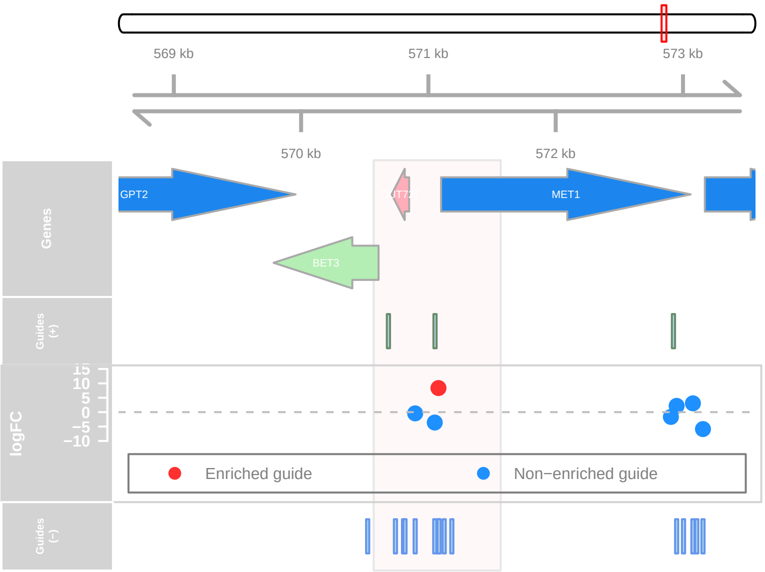

CUT480 on chr4

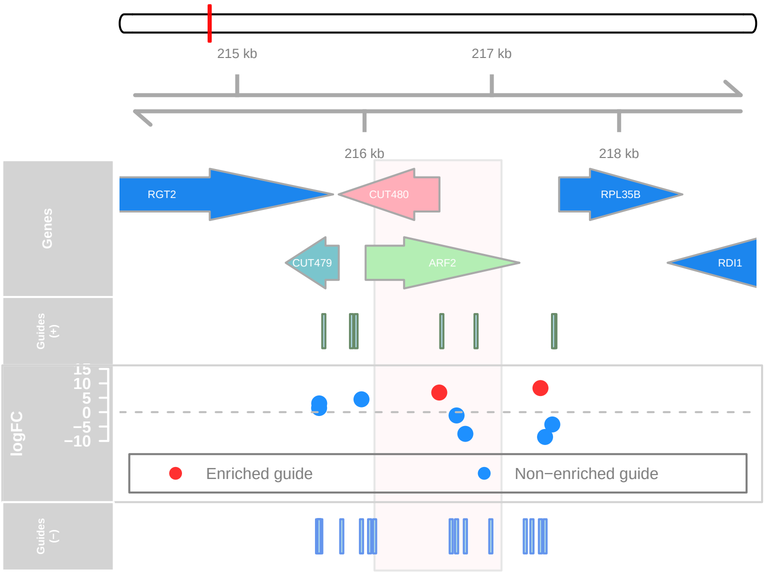

SUT128 on chr7

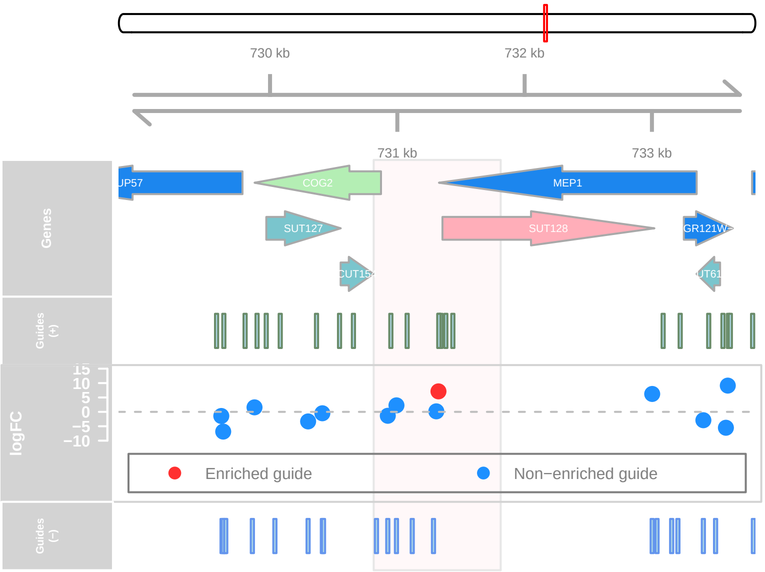

Supplement: ESI 2 [file EMS184631-supplement-ESI_2.pdf]
