# Supplementary material for: Large scale microfluidic CRISPR screening for increased amylase secretion in yeast
Source: Lab Chip. Author manuscript; Available in PMC 2023 Aug 15. (PMC7614956; doi:10.1039/d3lc00111c)
Supplement: ESI 5 [file EMS184631-supplement-ESI_5.pdf]

Electronic Supplementary Material (ESI) for Lab on a Chip.
This journal is © The Royal Society of Chemistry 2023

Supplementary figures

Figure S1. Gene ontology enrichment plots by gene counts. GO enrichment for cellular components (A-B), biological processes (C-D) and molecular functions (E-F), for activation (left) and repression (right) screens based on the TopGO analysis, showing the top 10 enriched terms per category by gene count, with darker colours indicating a higher significance (< p-value).

Figure S2. Genes associated with endosomal, vacuolar and Golgi apparatus components. in the vicinity of enriched SUTs and CUTs from the activation screens, within a 1 kb interval of the guide. Genes associated with endosomal, vacuolar and Golgi apparatus components are shown in light green, remaining genes are shown in dark blue, enriched SUTs and CUTs are shown in pink while remaining SUTs and CUTs are shown in teal, the direction of arrows indicate the coding strand. The position of all guides from the library are shown by strand, green (+ strand) and blue (- strand), while guides identified in the screen are shown as filled circles, with blue circles indicating detected but non-significantly enriched guides, and red filled circles indicating significantly enriched guides. The light red rectangle defines the 1 kb interval centred on the guide associated with the enriched SUT or CUT, for which included genes were searched for enrichment.

Figure S3. Examples of genes in the vicinity of enriched SUTs and CUTs from the repression screens. Genes of interest are shown in light green, remaining genes are shown in dark blue, enriched SUTs and CUTs are shown in pink while remaining SUTs and CUTs are shown in teal, the direction of arrows indicate the coding strand. The position of all guides from the library are shown by strand, green (+ strand) and blue (- strand), while guides identified in the screen are shown as filled circles, with blue circles indicating detected but non-significantly enriched guides, and red filled circles indicating significantly enriched guides. The light red rectangle defines the 1 kb interval centred on the guide associated with the enriched SUT or CUT, for which included genes were searched for function.

Figure S4. Validation of amylase secretion - non significant results. Genes identified during the activation and repression screens and validated using over-expression (TRS20) or gene deletion (YDR262W), which did not result in significant increases in α-amylase secretion over time. The results are shown as total secreted α-amylase (blue) and secreted α-amylase per cell (red) for measurements after 4, 24, and 48 hours, relative to the baseline α-amylase secretion of the transformed empty vector for gene over-expression, or the background strain only for gene deletion.
